# Human acetyl-CoA carboxylase 2 expressed in silkworm *Bombyx mori* exhibits posttranslational biotinylation and phosphorylation

**DOI:** 10.1007/s00253-014-5715-6

**Published:** 2014-04-17

**Authors:** In-Wook Hwang, Yu Makishima, Tatsuya Kato, Sungjo Park, Andre Terzic, Enoch Y. Park

**Affiliations:** 1 Laboratory of Biotechnology, Integrated Bioscience Section, Graduate School of Science and Technology, Shizuoka University, 836 Ohya, Suruga-ku, Shizuoka, 422-8529 Japan; 2 Laboratory of Biotechnology, Department of Applied Biological Chemistry, Faculty of Agriculture, Shizuoka University, 836 Ohya, Suruga-ku, Shizuoka, 422-8529 Japan; 3 Laboratory of Biotechnology, Green Chemistry Research Division, Research Institute of Green Science and Technology, Shizuoka University, 836 Ohya Suruga-ku, Shizuoka, 422-8529 Japan; 4 Center for Regenerative Medicine, Mayo Clinic, 200 First Street SW, Rochester, MN 55905 USA; 5 Marriott Heart Disease Research Program, Division of Cardiovascular Diseases, Departments of Medicine, Molecular Pharmacology and Experimental Therapeutics, and Medical Genetics, Mayo Clinic, 200 First Street SW, Rochester, MN 55905 USA

**Keywords:** Human acetyl-CoA carboxylase 2 (hACC2), Phosphorylation/dephosphorylation, Lipid metabolism, Silkworm, *Bombyx mori* nucleopolyhedrovirus

## Abstract

Biotin-dependent human acetyl-CoA carboxylases (ACCs) are integral in homeostatic lipid metabolism. By securing posttranslational biotinylation, ACCs perform coordinated catalytic functions allosterically regulated by phosphorylation/dephosphorylation and citrate. The production of authentic recombinant ACCs is heeded to provide a reliable tool for molecular studies and drug discovery. Here, we examined whether the human ACC2 (hACC2), an isoform of ACC produced using the silkworm BmNPV bacmid system, is equipped with proper posttranslational modifications to carry out catalytic functions as the silkworm harbors an inherent posttranslational modification machinery. Purified hACC2 possessed genuine biotinylation capacity probed by biotin-specific streptavidin and biotin antibodies. In addition, phosphorylated hACC2 displayed limited catalytic activity whereas dephosphorylated hACC2 revealed an enhanced enzymatic activity. Moreover, hACC2 polymerization, analyzed by native page gel analysis and atomic force microscopy imaging, was allosterically regulated by citrate and the phosphorylation/dephosphorylation modulated citrate-induced hACC2 polymerization process. Thus, the silkworm BmNPV bacmid system provides a reliable eukaryotic protein production platform for structural and functional analysis and therapeutic drug discovery applications implementing suitable posttranslational biotinylation and phosphorylation.

## Introduction

Acetyl-CoA carboxylases (ACCs) are biotin-dependent enzymes catalyzing the production of malonyl-CoA from acetyl-CoA, a critical metabolic intermediate in lipid metabolism (Brownsey et al. 2006; Kim 1997; Saggerson 2008; Tong 2013; Wakil and Abu-Elheiga 2009). Two different isoforms of ACC, ACC1 and ACC2, partake in lipid metabolism in humans and mammals (Abu-Elheiga et al. 1995, 1997; Ha et al. 1996). ACC1, encoded by *ACACA*, predominantly exists in the cytosol of lipogenic organs such as adipose tissue and liver where malonyl-CoA functions as a substrate for long chain fatty acids synthesis. In contrast, *ACACB*-encoded ACC2 is associated with the outer membrane of mitochondria in oxidative tissues such as the heart, liver, and skeletal muscle where malonyl-CoA is utilized as a negative regulator of fatty acid oxidation. Due to the bifunctional roles in catabolic and anabolic metabolism, ACC functions as a bioenergetics controller to promote stem cell function and tissue regeneration to regulate lipid homeostasis (Folmes et al. 2013; Fullerton et al. 2013; Knobloch et al. 2013; Park et al. 2013). Moreover, ACC2 knockout demonstrates anti-obesity effects and prevention of cardiac remodeling (Abu-Elheiga et al. 2001, 2003; Kolwicz et al. 2012). Therefore, ACC activity regulation has been recognized as an attractive therapeutic target for dysregulated lipid metabolism such as obesity, diabetes, cancer, and cardiovascular disease (Tong and Harwood 2006).

Eukaryotic ACCs, unlike prokaryotic ACCs composed of three separate functional proteins, comprises three distinctive functional domains, including a biotin carboxylase (BC) domain, a biotin carboxyl carrier protein domain (BCCP), and a carboxyltransferase (CT) domain, to carry out multiple functions. Integral in catalysis is biotin, a prosthetic group attached to lysine residue within the BCCP domain (Bianchi et al. 1990; Cronan and Waldrop 2002; Tanabe et al. 1975; Tong 2013). The BC domain catalyzes the Mg-ATP-dependent carboxylation of biotin to form carboxybiotin using bicarbonate as the CO_2_ donor (reaction 1). Then, carboxybiotin is transferred to the CT domain mediating transfer of the carboxyl group from carboxybiotin to acetyl-CoA to form malonyl-CoA (reaction 2). Besides these core catalytic reactions, ACC activities are allosterically regulated by multiple factors including phosphorylation/dephosphorylation and citrate (Beaty and Lane 1983a; Brownsey et al. 2006; Ha et al. 1994; Meredith and Lane 1978; Munday et al. 1988; Wojtaszewski et al. 2003). Thus, ACC with posttranslational biotinylation and properly regulated by allosteric modulators is essential in evaluating the full functionality of multistep reactions to unfold its functional mechanisms and systematic inhibitor discovery efforts.

1 $$ \mathrm{ATP}-{{\mathrm{Mg}}_2}^{+}+{{\mathrm{HCO}}_3}^{-}+\mathrm{ACC}-\mathrm{biotin}\leftrightarrow \mathrm{ACC}-\mathrm{biotin}-{{\mathrm{CO}}_2}^{-}+\mathrm{ADP}+{\mathrm{P}}_{\mathrm{i}} $$

2 $$ \mathrm{ACC}-\mathrm{biotin}-{{\mathrm{CO}}_2}^{-}+\mathrm{acetyl}-\mathrm{CoA}\leftrightarrow \mathrm{malonyl}-\mathrm{CoA}+\mathrm{ACC}-\mathrm{biotin} $$

The baculovirus expression system has been considered as the most efficient eukaryotic heterologous protein expression system as the host insect cells can implement foolproof posttranslational modifications similar to higher eukaryotes (Kost et al. 2005; Possee 1997). Two types of baculovirus expression systems, i.e., *Autographa california* multiple nucleopolyhedrovirus (AcMNPV) and *Bombyx mori* nucleopolyhedrovirus (BmNPV) systems, have been widely used (Kost et al. 2005; Maeda 1989). We developed a BmNPV bacmid, an *Escherichia coli* and *B. mori* hybrid shuttle vector, to expedite the heterologous protein production platform without construction and amplification of viruses in *B. mori* culture cells as recombinant BmNPV DNA can be directly injected into silkworm pupae or larvae (Hiyoshi et al. 2007; Motohashi et al. 2005; Park et al. 2008a). Using the BmNPV bacmid system, intracellular, extracellular, and membrane proteins have been successfully generated with proper folding and posttranslational modifications (Kato et al. 2010, 2012; Otsuki et al. 2013).

Here, we examined whether the recombinant human ACC2, produced using the silkworm BmNPV bacmid-based approach, secures proper posttranslational modifications to fulfill the essential catalysis and allosteric modulation. We report that ACC2, demonstrating consistent catalytic activities with proper posttranslational biotinylation and phosphorylation, is regulated by allosteric modulation. Thus, silkworm-based BmNPV system provides a reliable large-scale protein production platform for structural and function studies as well as drug discovery applications implementing essential posttranslational modifications.

## Materials and methods

### Construction of recombinant hACC2 BmNPV bacmid

The human ACC2 (2,548 amino acids) is associated with the mitochondria membrane through N-terminal 148 hydrophobic amino acids classified as mitochondrial attachment and target sequences (Tong 2013). To increase the solubility of recombinant human ACC2 (hACC2), these hydrophobic residues were excluded using polymerase chain reaction (PCR) with the following set of primers, 5′-GCCGTCGACATGTCCAAAGAAGACAAGAAGCAG-3′ (forward), 5′-GCTCTAGATTACTTGTCATCGTCATCCTTGTAGTCGGTGGAGGCCGGGCTGTCCATG-3′ (reverse), and designated as Δ148aa-hACC2. The PCR cycle was performed following 30 cycles of denaturation at 94 °C for 30s, annealing at 55 °C for 30s, and extension at 72 °C for 7 min, followed by a final extension at 72 °C for 10 min. The complementary DNA of human ACC2 from Mammalian Gene Collection (Thermo Scientific, Pittsburgh, PA, USA) was used as a template. The resultant PCR product was digested with *Sal*I and *Xba*I followed by purification with a GFX™ PCR DNA and Gel Band Purification Kit (GE Healthcare, Amersham, UK). The purified DNA fragment was ligated into pFastbac 1 vector, which was transformed into *E. coli* competent DH5α cells (Invitrogen, Carlsbad, CA, USA) and cultured on a solid LB medium containing 100 μg/mL of ampicillin at 37 °C for 18 h to generate recombinant plasmid. The plasmid containing human Δ148aa-hACC2 gene was isolated and identified by DNA sequencing. Finally, *E. coli* BmDH10bac-*CP* ^–^-*Chi* ^–^ competent cells containing the cysteine proteinase- and chitinase-deficient BmNPV bacmid (Park et al. 2008a) were transformed with the pFastbac1-Δ148aa-hACC2 and cultured on a solid LB medium containing 50 μg/mL of kanamycin, 7 μg/mL of gentamycin, 10 μg/mL of tetracycline, 40 μg/mL of isopropyl β-d-1-thiogalactopyranoside (IPTG), and 100 μg/mL of 5-bromo-4-chloro-3-indolyl-4-galactoside (X-Gal) (Takara Bio Inc., Otsu Shiga, Japan) at 37 °C for 18 h. The bacmid containing BmNPV-Δ148aa-hACC2 was isolated from white positive colonies.

### Expression and purification of recombinant hACC2 in silkworm

Silkworm pupae were used for the expression of recombinant Δ148aa-hACC2 as a bioreactor. To produce recombinant protein in pupae, 10 μg of BmNPV-Δ148aa-hACC2 bacmid DNA was directly injected with DMRIE-C reagent (Invitrogen) into the dorsal of pupae. The injected pupae were reared at 27 °C for 6–7 days, and stored at –80 °C until further analysis. Protein purification was carried out at 4 °C to minimize aggregation and protease activity. Five pupae were homogenized in 10 mL of lysis buffer (50 mM Tris-HCl, 150 mM NaCl, pH 7.4, and 0.1 % TritonX-100) containing an EDTA-free protease inhibitor tablet (Roche, Mannheim, Germany) using a homogenizer (GLH-115, Yamato, Tokyo, Japan). Cell debris was removed by pelleting through centrifugation at 12,000 × *g* for 30 min. The supernatant was filtered using a 0.45-μm syringe filter and loaded onto a 500-μL of Anti-FLAG M2 antibody Affinity Gel (Sigma-Aldrich, St. Louis, MO, USA) pre-equilibrated with equilibration buffer (50 mM Tris-HCl, 150 mM NaCl, pH 7.4 and 0.02 % TritonX-100). The column was washed with 2.5 mL of equilibration buffer and eluted with elution buffer (100 μg/mL FLAG peptide in 50 mM Tris-HCl and 150 mM NaCl, pH 7.4). The eluted Δ148aa-hACC2 was collected and concentrated using a 100 K Amicon Ultra centrifugal filter (Millipore, Billerica, MA, USA).

### Confirmation of biotinylation and phosphorylation by Western blotting

The posttranslational biotinylation and phosphorylation of purified Δ148aa-hACC2 were measured by Western blotting analysis. Prior to electrophoresis, purified sample was boiled for 5 min at 95 °C with protein denaturing buffer (Nacalai Tesque, Kyoto, Japan). Samples were electrophoresed in a 5 % SDS-PAGE gel with the Mini-protean system (Bio-Rad, Hercules, CA, USA) at 150 V for 45–60 min in Tris-glycine buffer (25 mM Tris, 250 mM glycine, pH 8.3, and 0.1 % SDS). The separated proteins on a SDS-PAGE gel were transferred to PVDF membranes (GE Healthcare) by electroblotting on a wet blotter (Bio-Rad) at 15 V for 1 h. To detect the purified Δ148aa-hACC2 and their biotinylation and phosphorylation, several specific antibodies were used. A mouse anti-FLAG antibody (Wako Pure Chem. Ind. Ltd., Osaka, Japan) was used to detect purified Δ148aa-hACC2 as a primary antibody. A monoclonal anti-phosphoserine antibody (Sigma-Aldrich) was used for phosphorylation detection as a primary antibody. An anti-mouse IgG-HRP (GE Healthcare) was used for above both cases as a secondary antibody. A goat anti-biotin antibody (Abcam, Cambridge, MA, USA) and streptavidin HRP conjugate (Thermo Scientific, Rockford, IL, USA) were used for biotinylation detection as a primary antibody. A rabbit anti-goat IgG-HRP (Santa Cruz Biotechnology, Santa Cruz, CA, USA) and an anti-mouse IgG-HRP were used as a secondary antibody.

### Dephosphorylation of Δ148aa-hACC2

Dephosphorylation was carried out using Lambda protein phosphatase (Lambda PP; New England Biolabs, Ipswich, MA, USA). The purified Δ148aa-hACC2 was incubated with 0.5 μL of Lambda PP, 1X NEBuffer for protein metallophosphatases (PMP) and 1 mM MnCl_2_ at 30 °C for 0, 1, 3, 6 h. Sterilized water instead of Lambda PP was used as negative control. Dephosphorylation was confirmed by Western blot using a monoclonal anti-phosphoserine antibody (Sigma-Aldrich) produced in mouse and an anti-mouse IgG-HRP (GE Healthcare). The activity of dephosphorylated Δ148aa-hACC2 was determined using ACC assay.

### Acetyl-CoA carboxylase assay

To measure ACC activity, 4 μL of purified Δ148aa-hACC2 was incubated with 36 μL of reaction buffer (50 mM of HEPES, pH 7.4, 5 mM of NaHCO_3_, 10 mM of MgCl_2_, 10 mM of sodium citrate, 0.5 % of DMSO, 4 mM of ATP, and 0.4 mM of acetyl-CoA) at 37 °C. The reaction was terminated by addition of 4 μL of 100 % trichloroacetic acid. The produced phosphate during the reaction was determined using a SensoLyte® MG Phosphate Assay Kit (AnaSpec, Fremont, CA, USA) by measuring the absorbance at 655 nm. Protein concentration was determined using BCA protein assay kit (Thermo Scientific).

### Citrate-induced polymerization of Δ148aa-hACC2

In order to confirm the allosteric regulation of purified Δ148aa-hACC2 from silkworm, citrate-induced polymerization was evaluated. The purified Δ148aa-hACC2 was incubated with 50 mM of HEPES (pH 7.4), 1 mM of dithiothreitol (DTT), and different concentration of citrate at 37 °C for 30 min. The polymerization was evaluated by Native-PAGE and Western blotting. Purified proteins were prepared in a non-denaturing sample buffer (Native sample buffer, Bio-Rad). Samples were electrophoresed in a 5 % Native-PAGE (without SDS) with the Mini-protean system (Bio-Rad) at 150 V for 45–60 min in Tris-glycine buffer (25 mM Tris and 250 mM glycine, pH 8.3). Next, Western blotting protocol was used as described above. The antibodies for detecting polymerized Δ148aa-hACC2 were same as used for detecting purified Δ148aa-hACC2. The activity of Δ148aa-hACC2 by citrate concentration-dependent polymerization was determined by ACC assay.

### Atomic force microscopy

Nanoscale AFM imaging was employed to investigate the dynamic forms polymers due to allosteric regulation of hACC2 by citrate. The Δ148aa-hACC2 was incubated with or without 15 mM citrate for 20 min at 37 °C. The resultant mixtures were placed on the freshly cleaved mica surface and incubated for several hours in a moisture chamber. After washing with water and drying under nitrogen, the samples were subjected to tapping mode AFM imaging on the Nanoscope IV PicoForce Multimode AFM, equipped with an E-scanner and a rectangular-shaped silicon cantilever (Bruker, Madison, WI, USA) with a 42-N/m spring constant and a resonant frequency of ~300 kHz at ambient environment (Park et al. 2008b; Park and Terzic 2010). Images (512 × 512 pixels/image) were collected from each sample with maximum image size of 5 × 5 μm and analyzed using the Nanoscope Version 6.13 software (Bruker).

## Results

### Expression and purification of recombinant Δ148aa-hACC2

**Fig. 1:**
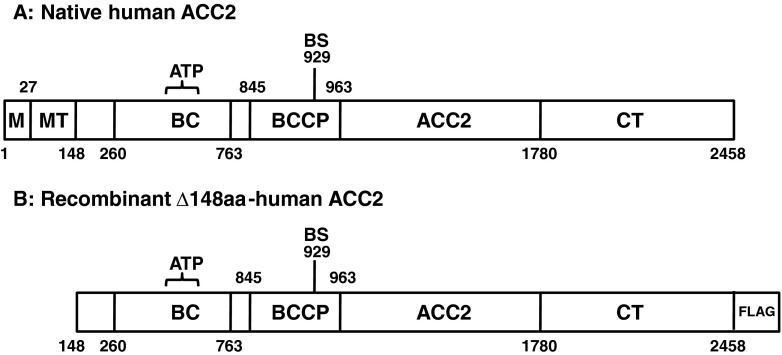
A schematic presentation of human ACC2 domains. **a** Native hACC2. *ACC2*, human acetyl-CoA carboxylase 2; *ATP*, ATP-grasp domain; *BC*, biotin carboxylase domain; *BCCP*, biotin carboxyl carrier protein domain; *BS*, biotinylation site; *CT*, carboxyltransferase domain; *M*, membrane attachment domain; *MT*, mitochondria targeting sequence. (**b**) Recombinant Δ148aa-hACC2. N-terminal 148 amino acids were deleted for increasing the solubility. FLAG was tagged at its C-terminus for affinity purification

Human ACC2 is a large polypeptide comprised of a mitochondrial attachment domain, a mitochondrial target sequence domain, a biotin carboxylase domain, a biotin carboxyl carrier protein domain, and a carboxyltransferase domain (Fig. 1a) (Bianchi et al. 1990; Tanabe et al. 1975; Tong 2013). Biotin is covalently attached to lysine within BCCP domain through posttranslational modification, and several serine residues are phosphorylated by protein kinases (Beaty and Lane 1983a; Brownsey et al. 2006; Ha et al. 1994; Meredith and Lane 1978). We deleted the N-terminal 148 hydrophobic amino acids to enhance the solubility of heterologous protein, which retains core functional modules (Fig. 1b). In particular, to prevent protease activity and liquefaction of heterologous proteins in silkworm-based expression system, *E. coli* BmDH10bac-*CP* ^–^-*Chi* ^–^ competent cells were employed.

**Fig. 2:**
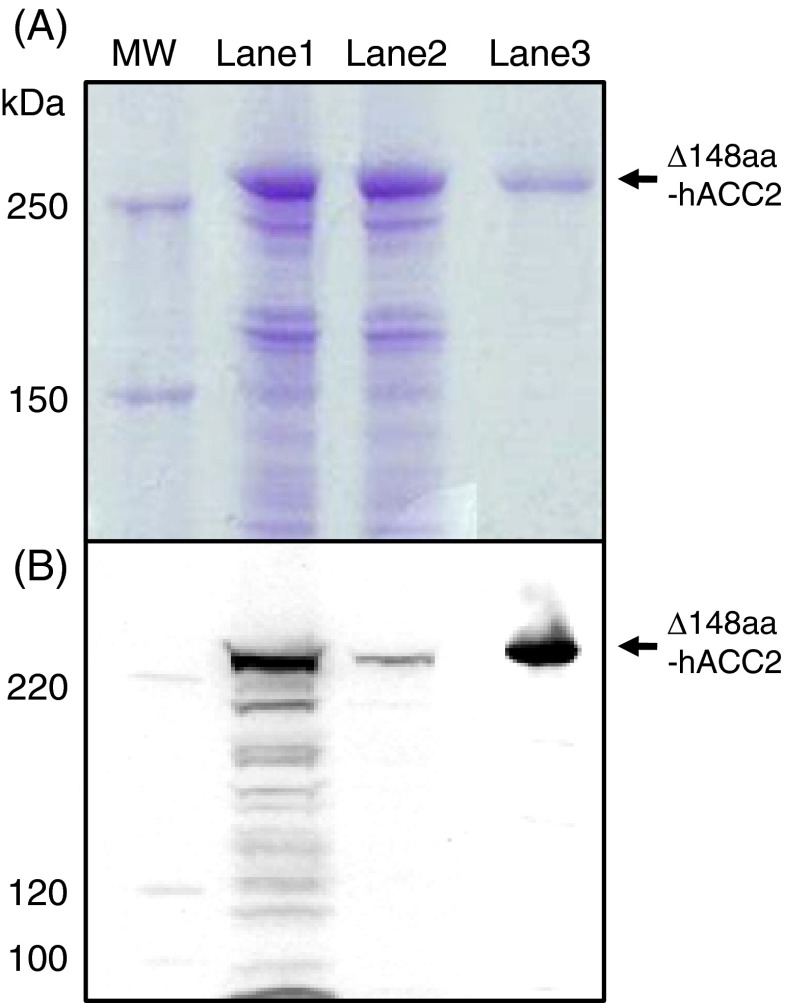
The expression of recombinant Δ148aa-hACC2 was confirmed by analysis of SDS-PAGE (**a**) and Western blot (**b**). *MW*, molecular weight markers; *Lane 1*, protein extracts after infection; *Lane 2*, flow through during FLAG-tag purification; *Lane 3*, purified and concentrated Δ148aa-hACC2. An anti-FLAG M2 antibody and an anti-mouse IgG-HRP were used to detect purified Δ148aa-hACC2

Recombinant Δ148aa-hACC2 with a C-terminal FLAG tag was purified using an anti-FLAG M2 affinity gel column. Eluted with FLAG peptides, the enriched protein migrated to ~260 kDa, a predicted molecular weight, on SDS-PAGE based on comparison with molecular weight markers (Fig. 2a). Western blot analysis using a FLAG-specific antibody confirmed the expression of hACC2 (Fig. 2b). In addition, the yield of final purified Δ148aa-hACC2 was 495 μg/pupa. This pupae-based recombinant protein expression provided a high yield of purified Δ148aa-hACC2 compared to expression in silkworm larvae (150 μg/larva) (Park et al. 2013). The purified hACC2 displayed significant homogeneity on SDS-PAGE and Western blot analysis, thereby further functional analysis was carried out using this enriched Δ148aa-hACC2.

### Biotinylation of Δ148aa-hACC2

**Fig. 3:**
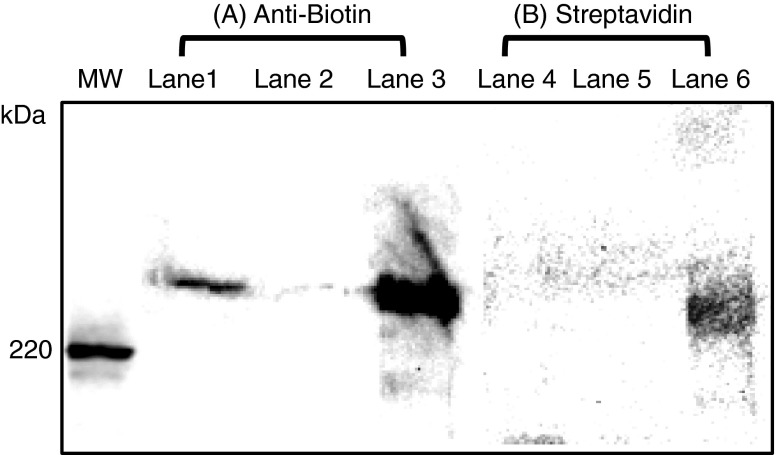
The purified Δ148aa-hACC2 possesses posttranslational biotinylation confirmed by Western blot analysis using an anti-biotin antibody (*A*) and streptavidin HRP conjugate (*B*). *MW*, molecular weight markers; *Lanes 1 and 4*, protein extracts after infection; *Lanes 2 and 5*, flow through during FLAG-tag purification; *Lanes 3 and 6*, purified and concentrated Δ148aa-hACC2. An anti-biotin antibody and a streptavidin HRP conjugate were used as primary antibodies. A rabbit anti-goat IgG-HRP and an anti-mouse IgG-HRP were used as secondary antibodies

The posttranslational modification with biotin in ACC2 is essential to implement catalytic function. The biotin binding residue in human ACC2 has not been clearly identified, yet structural studies using nuclear magnetic resonance suggests that biotin is attached to lysine 929 within a BCCP domain (Lee et al. 2008). The biotinylation of Δ148aa-hACC2 from the silkworm was analyzed by Western blotting using an anti-biotin antibody. To further validate biotinylation of the Δ148aa-hACC2, streptavidin HRP conjugate was employed to detect the biotin group as streptavidin is known to interact with biotin with very high affinity. Although the hACC2-anti-biotin band was detected more intensely than the streptavidin bound band (Fig. 3), biotin-specific detection using two different methods confirmed hACC2 biotinylation. Collectively, without additional supplement of biotin to generate biotinylated ACC observed in *Trichoplusia ni* cells (Kim et al. 2007), silkworm enables to produce biotinylated heterologous proteins.

### Phosphorylation and dephosphorylation of Δ148aa-hACC2

Adenosine monophosphate-activated protein kinase (AMPK)-mediated phosphorylation is other layer of posttranslational modification to allosterically regulate ACC catalytic function. Phosphorylation inactivates ACC catalytic activity whereas dephosphorylation activates the enzymatic function. Notably, phosphorylation of Ser222 in hACC2 (Ser212 in mouse ACC2) has been recognized as a vital process for homeostatic lipid metabolism (Fullerton et al. 2013; Wakil and Abu-Elheiga 2009). Consistent with these findings, the crystal structure of biotin carboxylase domain of hACC2 has revealed that the phosphorylation of Ser222 disrupts the polymerization of ACC2, a widely recognized mechanism in modulating catalytic function (Cho et al. 2010; Lee et al. 2008).

**Fig. 4:**
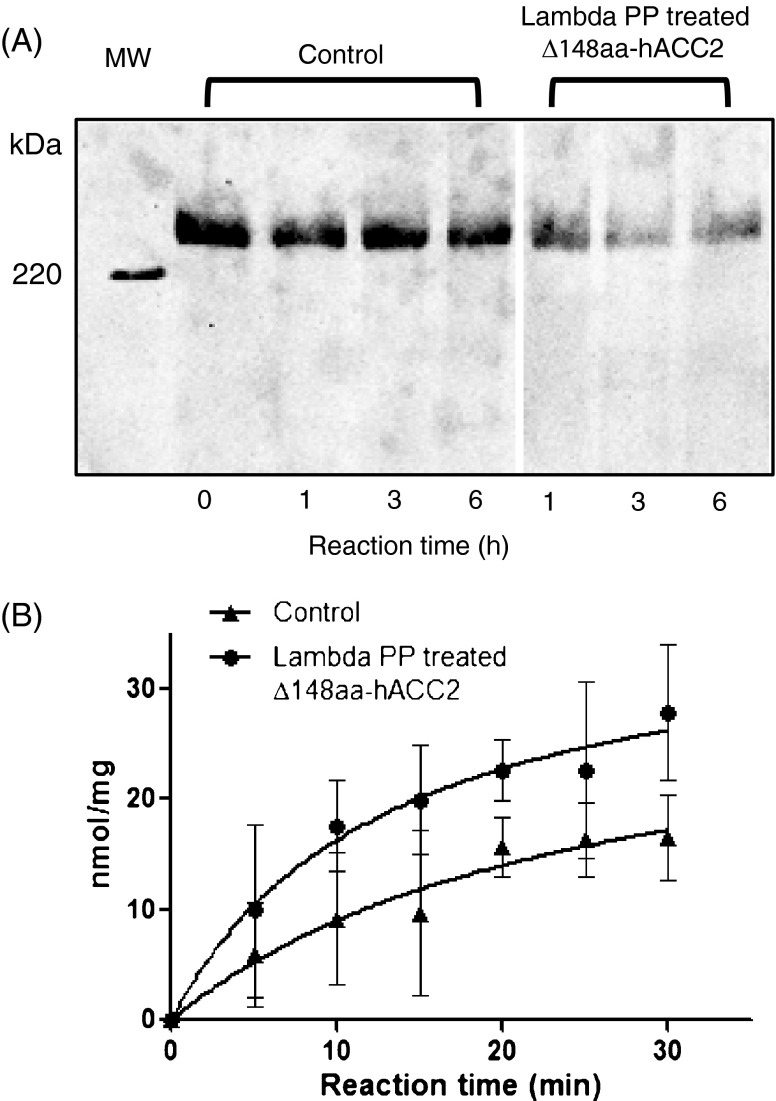
Dephosphorylation of purified Δ148aa-hACC2 influences catalytic function. **a** Dephosphorylation of Δ148aa-hACC2 treated with Lambda PP was assessed by Western blotting using a monoclonal anti-phosphoserine antibody produced in mouse and an anti-mouse IgG-HRP. **b** Lambda PP treated Δ148aa-hACC2 enhanced catalytic activity

We evaluated posttranslational phosphorylation of recombinant Δ148aa-hACC2, and then whether the phosphorylated protein could be effectively dephosphorylated accompanying the changes of catalytic function. Western blotting analysis using a monoclonal anti-phosphoserine antibody demonstrated the phosphorylation of Δ148aa-hACC2 purified from silkworm pupae (Fig. 4a). The addition of Lambda protein phosphatase, a Mn^2+^-dependent dephosphorylation enzyme, gradually decreased the phosphorylation compared with control (Fig. 4a), yet dephosphorylation was not completely achieved in Δ148aa-hACC2 up to 6 h incubation. This finding suggests that some of phosphorylation sites in full-length hACC2 could be readily inaccessible by Lambda protein phosphatase unlike isolated functional domains such as a biotin carboxylase domain (Kwon et al. 2013).

The effect of dephosphorylation was assessed by measuring the catalytic function of Δ148aa-hACC2 (Fig. 4b). Δ148aa-hACC2 was treated with and without Lambda protein phosphatase for 2 h, and then catalytic activity was measured as a function of incubation time. Purified Δ148aa-hACC2 with indigenous posttranslational phosphorylation provided a specific activity of 0.786 ± 0.229 nmol P_i_/mg/min (*n* = 6), whereas phosphatase treated Δ148aa-hACC2 protein yielded a specific activity of 1.336 ± 0.441 nmol P_i_/mg/min (*n* = 6), about 2-fold increase. These measurements are consistent with the findings observed in knock-in mice samples where Ser212 (mouse sequence) is replaced with alanine to ablate the critical serine phosphorylation (Fullerton et al. 2013).

### Allosteric activation of Δ148aa-hACC2 by citrate

Citrate-induced polymerization has been extensively employed to understand the regulatory mechanism of ACC, although the concentrations of citrate required for allosteric activation are much higher than that present at physiological locale (Beaty and Lane 1983a, b; Gregolin et al. 1966; Kim et al. 2010). Upon incubation with citrate, ACC polymerizes into filamentous structures containing 10–20 protomer units with increased functional activity (Kim et al. 2007; Locke et al. 2008). Regardless of biological significance of citrate in ACC regulation, the citrate binding sites have not been identified.

**Fig. 5:**
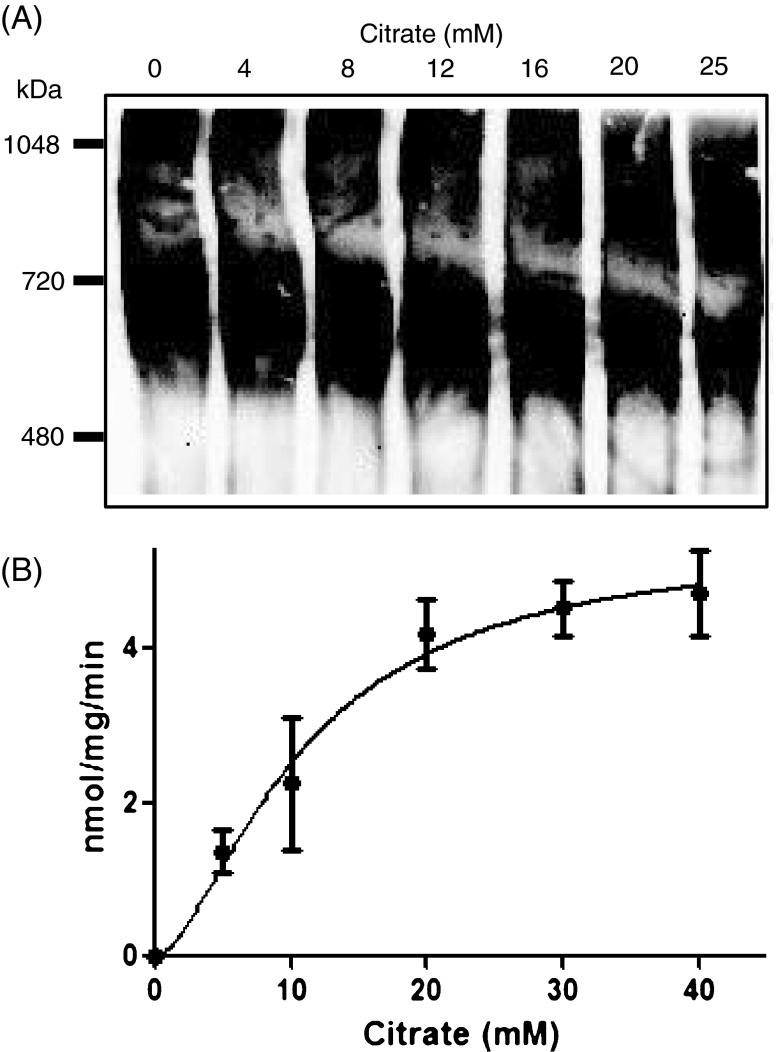
Polymerization and enzyme activities of Δ148aa-hACC2 were modulated by citrate concentration. **a** Degree of polymerization of Δ148aa-hACC2 by different concentration of citrate (0, 4, 8, 12, 16, 20, and 25 mM). The polymerization was confirmed using Native-PAGE. **b** Enzymatic activities by citrate concentration. All data are means ± S.D. from three separate experiments

The citrate-induced allosteric activation of Δ148aa-hACC2 was investigated by measuring the modulation of structural and functional properties. Incubation of Δ148aa-hACC2 with citrate generated the formation of high molecular weight polymers detected on Native-PAGE, which was increased with rising citrate concentrations (Fig. 5a). This polymerization results indicate that Δ148aa-hACC2 derived from pupae consists of dimers and tetramers based on comparison with molecular weight markers, and increased citrate concentrations led to tetramer production by decreasing dimers (Fig. 5a). Consistent with dose-dependent polymerization, the catalytic activity of Δ148aa-hACC2 was also enhanced with increasing citrate concentrations (Fig. 5b). Incubation of Δ148aa-hACC2 with 5, 10, and 20 mM citrate produced specific activity of 1.363 ± 0.279, 2.246 ± 0.870, and 4.186 ± 0.200 nmol P_i_/mg/min (*n* = 4), respectively. These values indicated that when the citrate concentration was increased by 2-fold, activities were also increased by about 2-fold in proportion to citrate concentration (Fig. 5b). Furthermore, when the concentration of citrate exceeds 20 mM, the activation curve follows a sigmoidal response, consistent with previous findings (Cheng et al. 2007).

**Fig. 6:**
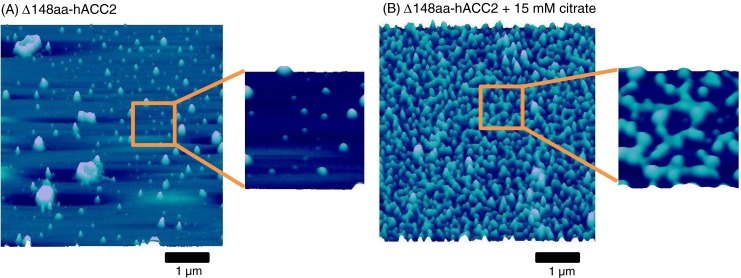
Atomic force microscopy nanoscale images of Δ148aa-hACC2. **a** Δ148aa-hACC2 alone. **b** Δ148aa-hACC2 with 15 mM citrate after 20-min incubation at 37 °C. Citrate induced the filaments formation

The structural changes of Δ148aa-hACC2 by citrate were also evaluated using high-resolution AFM at nanoscale resolution (Fig. 6). The purified Δ148aa-hACC2 without citrate showed almost homogeneous particle distribution. However, citrate addition to Δ148aa-hACC2 generated filamentous polymeric forms, significantly larger than Δ148aa-hACC2 alone. These findings not only support the formation of high molecular weight polymers observed in Native-PAGE but also validate that Δ148aa-hACC2 produced in silkworm possess full functionality with proper allosteric modulations.

## Discussion

Acetyl-CoA carboxylase is a multidomain and multifunctional protein working as an energetic controller in homeostatic lipid metabolism participating in fatty acid synthesis and fatty acid oxidation (Tong 2013; Wakil and Abu-Elheiga 2009). The catalytic function of ACC through a biotin prosthetic group can be allosterically regulated by multiple factors including posttranslational phosphorylation and dephosphorylation (Wakil and Abu-Elheiga 2009). In addition, tertiary level regulation of ACC with small acidic proteins, i.e., Spot14 and Mig12, has been recently identified (Colbert et al. 2010; Kim et al. 2010; Knobloch et al. 2013; Park et al. 2013). Particularly, due to the pathophysiological relevance of ACC2 in lipid metabolic syndrome associated with obesity, diabetes, cancer, and cardiovascular disease, ACC2 activity regulation has been considered as a candidate target for therapeutic interventions, which essentially requires authentic bioengineered recombinant proteins (Abu-Elheiga et al. 2001; Tong 2013; Tong and Harwood 2006). Here, we successfully produced with high fidelity human ACC2 using the silkworm BmNPV system armed with a proper posttranslational modification machinery. The heterologous ACC2 harbors all necessary posttranslational biotinylation and phosphorylation, vital to maintain functional integrity. Thus, the silkworm BmNPV bacmid system provides a reliable large-scale production platform for eukaryotic proteins required for posttranslational modifications.

Biotin is a water-soluble vitamin serving as a vital prosthetic group involved in five carboxylases in human (Zempleni et al. 2009). In hACC2, biotin is covalently linked to lysine 929 within a BCCP domain and forms carboxybiotin using bicarbonate as the CO_2_ donor. Following a large conformational change, the carboxyl group from carboxybiotin is transferred to acetyl-CoA to produce malonyl-CoA (Tong 2013). We revealed posttranslational biotinylation of functional recombinant hACC2 from silkworm, without any additional supplement of biotin under silkworm rearing conditions. We believe that this is the first demonstration of posttranslational biotinylation in proteins expressed in silkworm *B. mori*.

ACC phosphorylation/dephosphorylation is one of the allosteric regulatory mechanisms. Phosphorylation of ACC by AMP-activated protein kinase and cAMP-dependent protein kinase inhibits the enzymatic activity of ACC, whereas dephosphorylation activates the catalytic function (Munday et al. 1988). Although several phosphorylation sites have been identified, Ser212 (mouse sequence) phosphorylation was recently recognized as an integral process in activity modulation (Fullerton et al. 2013) where knock-in mice with substitution of Ser212 with alanine displayed an increased ACC2 activity. Consistent with this, we demonstrated about a 2-fold increased specific activity in dephosphorylated hACC2 compared to the phosphorylated counterpart, underscoring that the critical serine residue is fully accessible to phosphatase protein. ACC is also allosterically activated by citrate, which is a metabolic intermediate produced in mitochondrial tricarboxylic acid cycle. Although the concentrations of citrate required to increase the enzymatic activity of ACC are much higher than the physiological concentrations of citrate, citrate has been widely used to modulate ACC function (Beaty and Lane 1983a; Cheng et al. 2007; Thampy and Wakil 1988). We demonstrated herein citrate-induced hACC2 catalytic function enhancement and filamentous polymer formation. In summary, a multifunctional human ACC2 was successfully produced using silkworm-based protein expression system. Heterologous ACC2 was correctly folded with posttranslational biotinylation and phosphorylation, retaining catalytic activity and citrate-induced allosteric regulation. Moreover, silkworm demonstrated a high yield of recombinant ACC2 production. Thus, the silkworm-based BmNPV expression method equipped with a proper posttranslational modification machinery provides a large-scale eukaryotic protein production platform for structural and functional research particularly in the application of therapeutic drug discovery.
